# A Narrative Review of Innovative Responses During the COVID-19 Pandemic in 2020

**DOI:** 10.3389/ijph.2022.1604652

**Published:** 2022-12-08

**Authors:** Tzu-Chi Wu, Chien-Ta Bruce Ho

**Affiliations:** ^1^ Institute of Technology Management, National Chung-Hsing University, Taichung, Taiwan; ^2^ Department of Emergency Medicine, Show Chwan Memorial Hospital, Changua, Taiwan

**Keywords:** COVID-19, innovation, healthcare system, technological innovation, frugal innovation, repurposing, process innovation

## Abstract

**Objectives:** The coronavirus disease 2019 (COVID-19) pandemic presented unprecedented challenges to healthcare systems worldwide. While existing studies on innovation have typically focused on technology, health providers still only have a vague understanding of the features of emergency responses during resource exhaustion in the early stage of a pandemic. Thus, a better understanding of innovative responses by healthcare systems during a crisis is urgently needed.

**Methods:** Using content analysis, this narrative review examined articles on innovative responses during the COVID-19 pandemic that were published in 2020.

**Results:** A total of 613 statements about innovative responses were identified from 296 articles and were grouped under the following thematic categories: medical care (*n* = 273), workforce education (*n* = 144), COVID-19 surveillance (*n* = 84), medical equipment (*n* = 59), prediction and management (*n* = 34), and governance (*n* = 19). From the four types of innovative responses extracted, technological innovation was identified as the major type of innovation during the COVID-19 pandemic, followed by process innovations, frugal innovation, and repurposing.

**Conclusion:** Our review provides insights into the features, types, and evolution of innovative responses during the COVID-19 pandemic. This review can help health providers and society show better and quicker responses in resource-constrained conditions in future pandemics.

## Introduction

The outbreak of the coronavirus disease 2019 (COVID-19) that began in December 2019 in Wuhan, China, subsequently extended globally; as of October 2022, over 624 million confirmed cases and 6.5 million deaths have been reported [1]. The exponential growth of cases far exceeded the conventional abilities and resources of global healthcare systems [2]. As these demands continued to grow beyond the capacity of healthcare systems, health providers were forced to respond by temporarily suspending, modifying, or optimizing many routine processes and services, including medical care, chronic disease management, and workforce education [3]. To respond effectively to the crisis and narrow the gap between medical supply and demand, a variety of innovative responses were developed to facilitate healthcare in the prevailing resource-constrained conditions.

Innovation is defined as the product or outcome of a new idea, method, or device, and as a process that introduces something new [4]. Generally, innovation in healthcare is difficult because of the potential for obstructions caused by various forces, including industry players, funding, public policy, technology, and customers [5]. However, the pandemic created a new scenario that served as a catalyst for innovation and enabled innovative responses not only in terms of the introduction of new techniques but also for problem solving and even indispensable pillar in healthcare system transformation. The COVID-19 pandemic created an urgent need to develop innovations to combat resource constraints. However, innovations during crises are not always market-oriented or focused on customer needs; instead, they usually focus on creation of solutions and provision of adequate healthcare to patients in resource-constrained environments [6]. As a result, some of these innovative responses are temporary and only meant for problem-solving, while others may continue to evolve even after the crisis.

Undoubtedly, the volume and variety of innovative responses caused by the pandemic are extraordinary [7]. Although existing studies on innovations in the healthcare system have typically focused on technology, they do not cover all kinds of innovative responses, and the details of the technology applications remain unclear [8, 9]. Since pandemics are likely to reoccur, there is an urgent need to explore the innovative responses developed by healthcare systems during the COVID‐19 pandemic. Therefore, this study aimed to (1) identify the features of innovative responses in the healthcare system during the COVID-19 era, (2) explore innovative solutions and technological applications in terms of different thematic categories related to the healthcare system, and (3) explore the features and relationships among resource requirements, response timeframes, and innovation types.

The thematic categories suggested from systematic reviews used domains to comprehensively examine healthcare-specific innovative responses [10, 11]. This classification was designed to identify the resources necessary to prepare for and deal with current and future crises in the healthcare system [11]. For this study, we modified and classified six thematic categories: medical care, medical equipment, COVID-19 surveillance, workforce education, prediction and application, and governance (Table 1).

**TABLE 1:** Definitions of the six thematic categories (Taiwan, 2021).

Medical Care	This thematic category encompasses all healthcare areas from primary care to specialist care, acute medicine to non-urgent chronic diseases, and individuals affected by COVID-19
Medical Equipment	This thematic category includes medical resources such as ventilators and hospital spaces, and protective equipment such as masks
COVID-19 Surveillance	This domain includes the testing and diagnosis of COVID-19, contact tracing, and social distancing surveillance
Workforce Education	This domain includes education in the medical field, medical students, residents, or trainees in hospitals, medical workforce, and health providers
Prediction and Application	This domain focuses on the application of techniques and decision-making strategies to predict and manage outbreaks
Governance	This domain includes the reorganization of the healthcare system, interdisciplinary coordination and collaboration, strategies for daily clinical operations, and clinical process improvement

## Methods

This narrative review aimed to gain insights into innovative responses during the COVID-19 pandemic, and the review protocol was registered under the registration number INPLASY2021110102. Using a qualitative approach, this study employed social science theory to interpret and analyze the contents of multiple articles and categorize and understand healthcare thematic categories and innovation features during the COVID-19 pandemic. Content analysis can yield insights from articles, extract various themes and topics from the text, and measure the frequency of pre-identified targets [12]. Content analysis has the potential to be a useful method because knowledge of innovative responses in the healthcare system during the COVID-19 crisis is currently fragmented [13].

### Research Questions

To ensure that a large amount of the literature related to the topic of concern was captured, we posed the following research questions:

(1) What were the innovative responses to the pandemic in the healthcare system?
(2) What were the types of innovations used during the pandemic, and the features of and connections among innovations in the healthcare system?
(3) What were the different thematic categories pertaining to the innovative responses in the healthcare system?
(4) What were the features of the relationships among innovative responses, response timeframes, and resource requirements during the pandemic?

**FIGURE 1:**
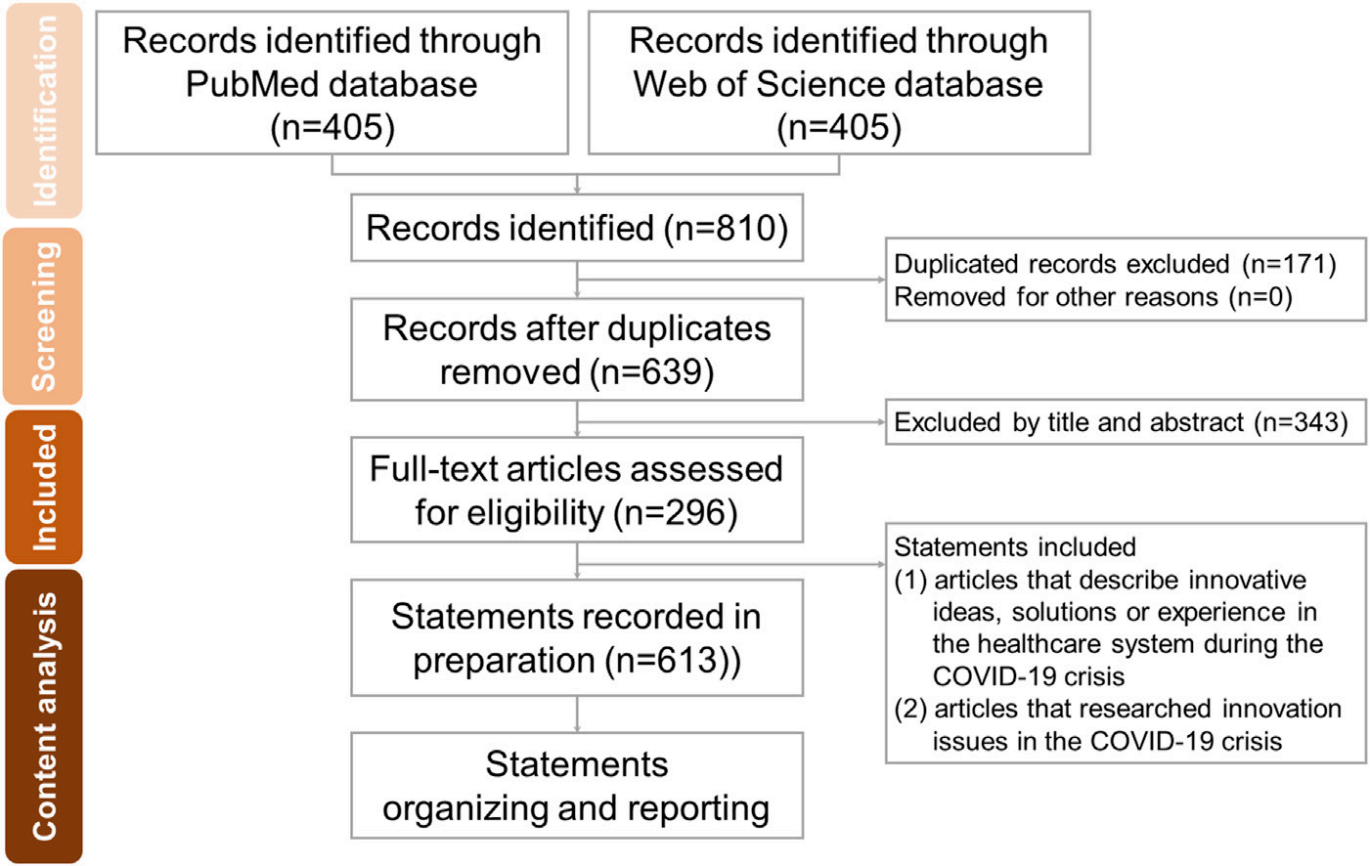
Flow diagram for the search and identification of included articles and content analysis (Taiwan, 2021).

The process of article collection and the approach used for content analysis is described in the following subsections (Figure 1).

### Search Strategy

On May 30, 2021, we performed a literature search on PubMed and Web of Science (WOS) for articles related to COVID-19 and innovations limited to the period from January 1 to December 30, 2020. Literature with unavailable full text was excluded. We searched the PubMed and WOS databases using the following search terms and database-appropriate syntax: “COVID-19” or “Novel virus” or “Coronavirus” or “2019-nCoV” or “SARS-CoV-2 virus and “Innovation.” The filters “Full text,” “English,” “from 2020 to 2020,” were applied in PubMed. The filters “2020” and “article” were applied in the WOS. A total of 405 articles each were identified through PubMed and WOS. Duplicated records (*n* = 171) were excluded, leaving 639 articles.

### Data Extraction and Inclusion Criteria

First, we defined innovation as “a new idea, product, method, service, or process introduced by the author of the article.” We selected (1) articles that described innovative responses in the healthcare system during the COVID-19 crisis and (2) articles that researched innovation issues in the COVID-19 crisis. We excluded (1) articles not focused on the COVID-19 pandemic, (2) articles related to vaccine and medicine development (e.g., novel compounds) and drug clinical trials, and [3] articles describing innovations in basic medical science, such as gene sequencing. The title, abstract, and conclusion of the included articles were manually reviewed. After applying the inclusion and exclusion criteria, 296 articles related to innovation and COVID-19 were collected.

### Research Approach and Process

First, one reviewer read all the articles and selected innovative episodes or solutions by manually highlighting the textual content. Details of innovative responses, including the technical and application fields, were recorded. An article could contain more than one innovation episode. Finally, 613 statements related to innovative responses were obtained and recorded. Next, the two reviewers organized the qualitative data through open coding, grouping, categorization, and abstraction [13]. Statements were grouped into higher-order categories by collapsing those that were similar. Finally, two reviewers named each category of innovation using content-characteristic words and extracted four types of innovation post discussion (1): technological innovation, (2) frugal innovation, (3) repurposing, and (4) process innovation.

The next step was to review each innovative response recorded to identify which of the six predefined domains and innovative response types it belonged to, and then analyze the data using inductive thematic analysis. To increase reliability, two authors went through the same process independently, and two coders agreed on 78% of the categorization. This difference was resolved after discussion and reassessment.

Additionally, keywords from 296 articles were used to generate a network graph of related terms, topics, and healthcare issues. Simultaneously, two strategic dimensions were used to evaluate the innovative responses (1): strategic stretching (referring to the degree of resource requirements, ranging from low to high), and (2) strategic horizon (referring to the response timeframe, ranging from short to long term). Short-term referred to the period during the crisis, and long-term referred to the period extending beyond the crisis.

## Results

**FIGURE 2:**
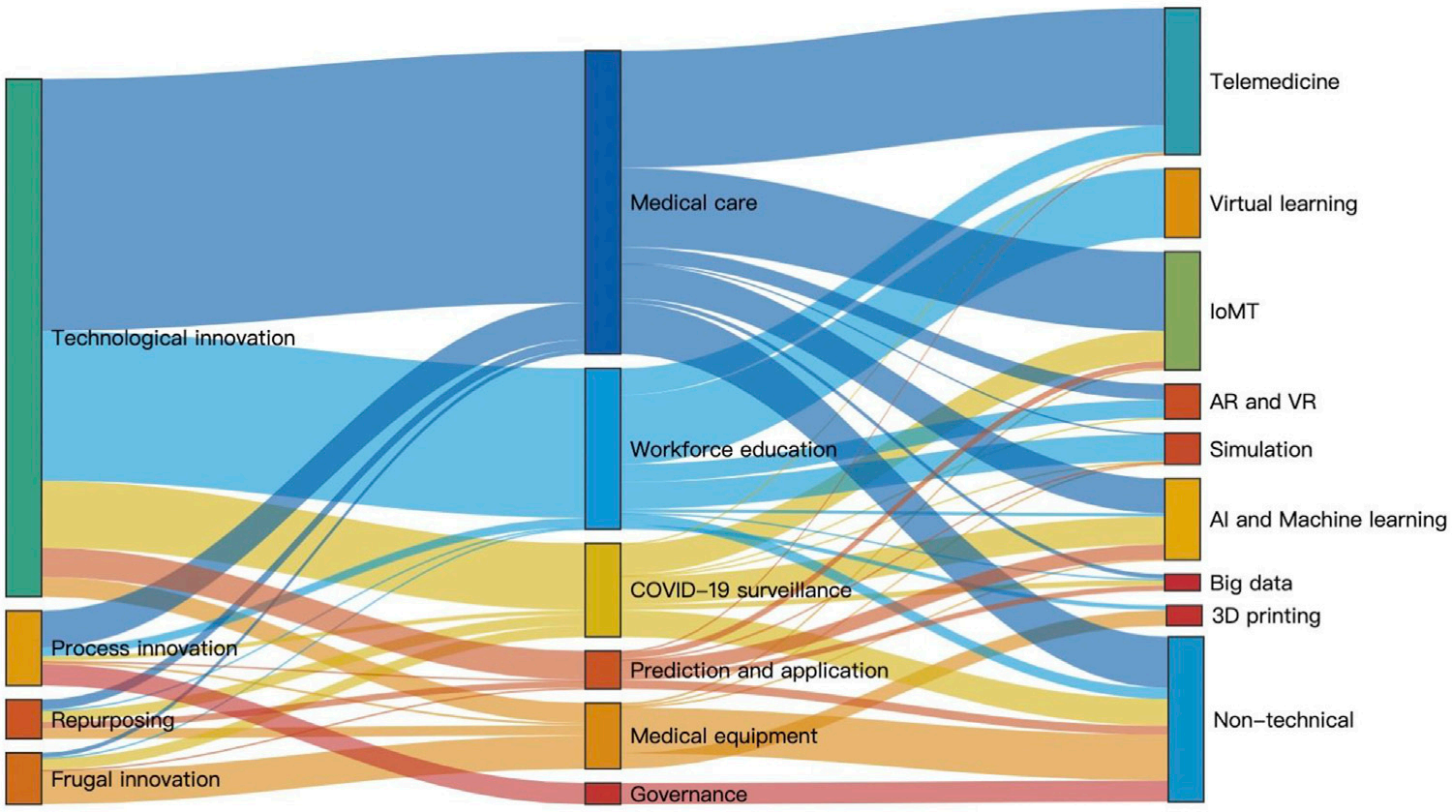
The Sankey diagram, with clusters by size, reveals different types of innovation (left) in the six thematic categories (middle) and the technology (right) in technological innovations (Taiwan, 2021).

A total of 613 statements on innovation were identified in the 296 articles. In descending order, the innovative responses from the 613 statements were grouped into six thematic categories: medical care (*n* = 273), workforce education (*n* = 144), COVID-19 surveillance (*n* = 84), medical equipment (*n* = 59), prediction and management (*n* = 34), and governance (*n* = 19). Innovation statements in the four types of extracted innovative responses were categorized as follows: technological innovations (*n* = 465), process innovations (*n* = 67), frugal innovation (*n* = 46), and repurposing (*n* = 35) (Figure 2).

### The Definitions of the Four Extracted Innovative Responses Were as Follows

On the basis of the WHO definition, we defined technological innovation as “the new method use of information or communications technology in support of health-related fields’’ [14]. Frugal innovation was defined as “a method or ability to do more with limited products or solutions” [32]. Repurposing was defined as “reuse of an existing innovation in a different context’’ [32]. Process innovation was defined as “a method, idea, or process to enhance internal production processes or change the way of service.’’ [15, 16].

In our review, technological innovation was the main response during the 2020 pandemic. Eight subtypes of application techniques were identified (1): telemedicine (*n* = 132), (2) IoMT (*n* = 106), (3) artificial intelligence and machine learning (*n* = 74), (4) big data (*n* = 15), (5) 3D printing (*n* = 18), (6) virtual learning (*n* = 62), (7) simulation (*n* = 27); and (8) augmented and virtual reality (*n* = 31). Among the thematic categories of medical equipment, 3D printing was the most important technological innovation. The application of big data analytics and IoMT mainly surfaced in the thematic categories of COVID-19 surveillance and prediction.

Frugal innovation was the main type of innovation within the thematic category of medical equipment. With resources such as ventilators, personal protective equipment (PPE), testing tools, and isolation rooms in short supply, frontline health providers sought cheap solutions to protect patients and themselves. In many countries, intubation boxes [31], collection booths [32], and other economical and easily assembled apparatuses made out of commonly available items were used to prevent the spread of infection [33], Similarly, using an extractor fan with a HEPA filter, temporary ICU rooms were created to address the shortage of ICU beds [34]. Moreover, to decrease PPE usage, nursing teams developed extended intravenous tubing that allows easy and rapid flow, effectively reducing back-and-forth entry into isolation rooms [35].

Process innovations mainly appear in the thematic categories of medical care and governance. First, healthcare providers attempted to limit face-to-face visits by adjusting processes. Since clinical processes were modified, surgical procedures were limited to only emergency or urgent cases [17]. In the thematic category of governance, the largest ICU in the UK, which managed a huge influx of critical patients and safely expanded the normal workings of a large critical care unit, improved its capacity and efficiency by breaking the whole process into smaller parts and redeploying staff [18].

Repurposing mainly occurred in the fields of medical care and medical equipment when the requirement of resources was relatively low, and the range of the timeframe was large. For example, point-of-care ultrasonography was repurposed to facilitate clinical decision-making during triage [32]. In addition, information about the efficacy of the antimalarial drugs chloroquine and hydroxychloroquine was repurposed to use them for COVID-19 treatment [33]. Moreover, many firms, such as Formula One teams, airplanes, and car manufacturers, used their capacity to repurpose their production lines to manufacture ventilators [35–37].

**FIGURE 3:**
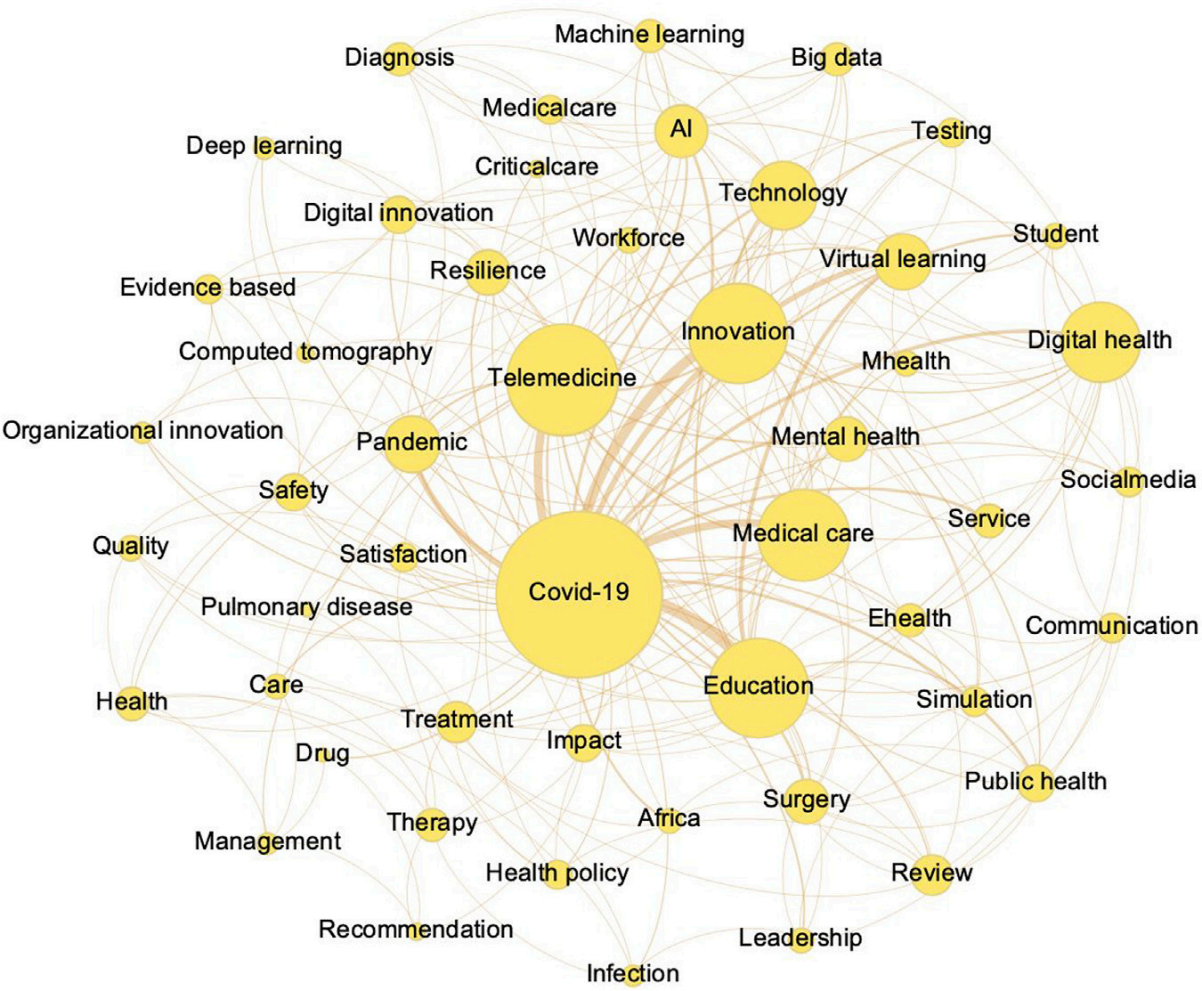
Network graph of key words of articles (Taiwan, 2021).

The network graph of the keywords of articles that appeared at least 15 times (Figure 3) revealed visualized clusters (keywords) and relationships between the nodes. Besides “COVID-19” and “innovation,” the main fields of application included medical care and education. Telemedicine appears to be the most widely used technique, followed by virtual learning and artificial intelligence (AI).

**FIGURE 4:**
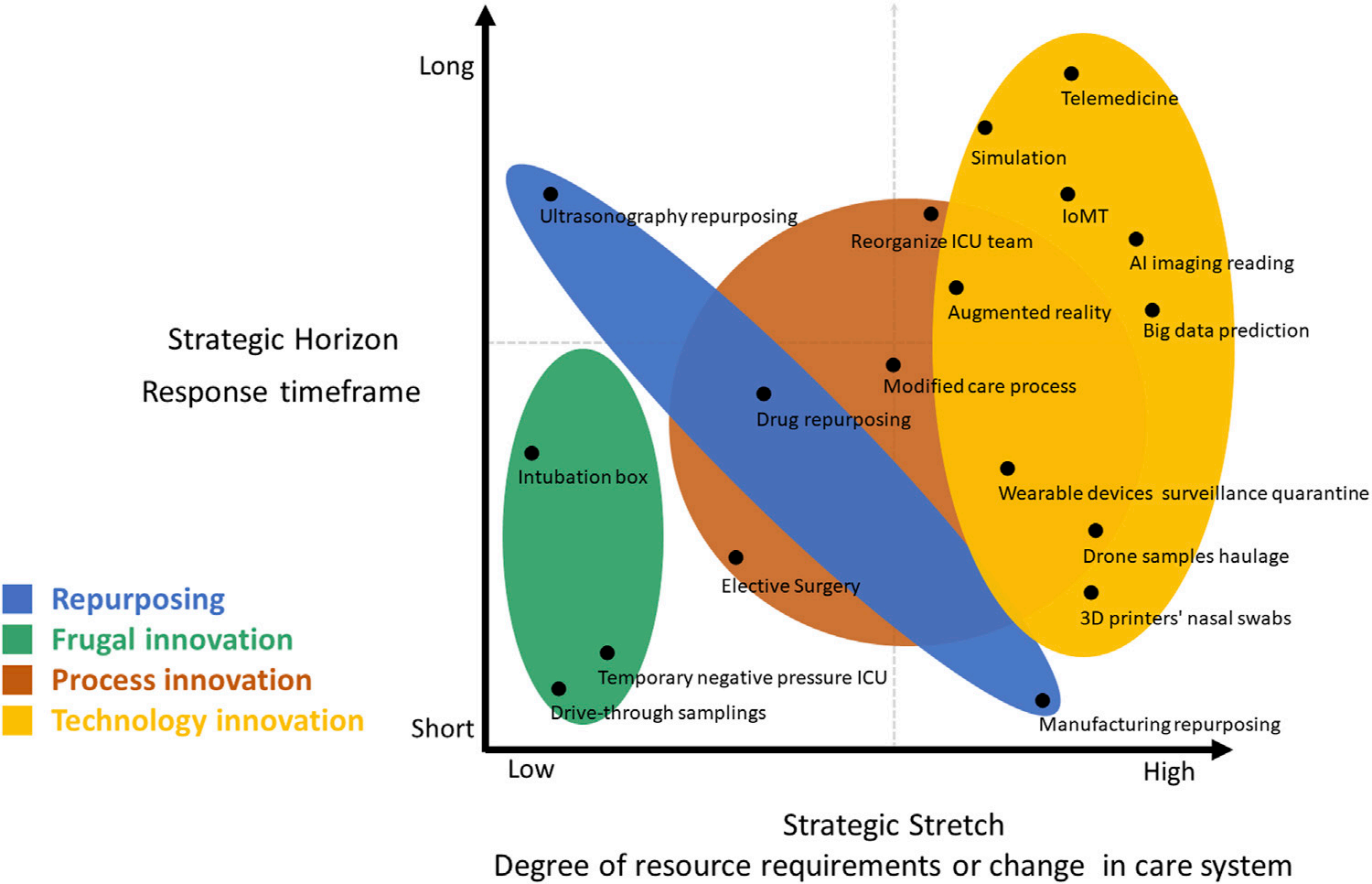
Innovation strategy and examples (Taiwan, 2021).

Through the strategy lens (Figure 4), we provided examples of innovations to reveal the timeframe and the degree of resource requirements. Innovations for frugality address immediate healthcare concerns without the intention to achieve long-term effects. The range of repurposing is large and depends on the type of repurposing. For instance, manufacturing repurposing for medical equipment requires major changes but only during the crisis period. Most technological innovations address not only the ongoing difficulties but also go beyond the immediate present, indicating a long-term effect. Process innovations generally take time; some processes will revert to their original form post-crisis, such as delayed elective surgery, while some may indicate more lasting changes, such as the reorganization of the healthcare system.

**FIGURE 5:**
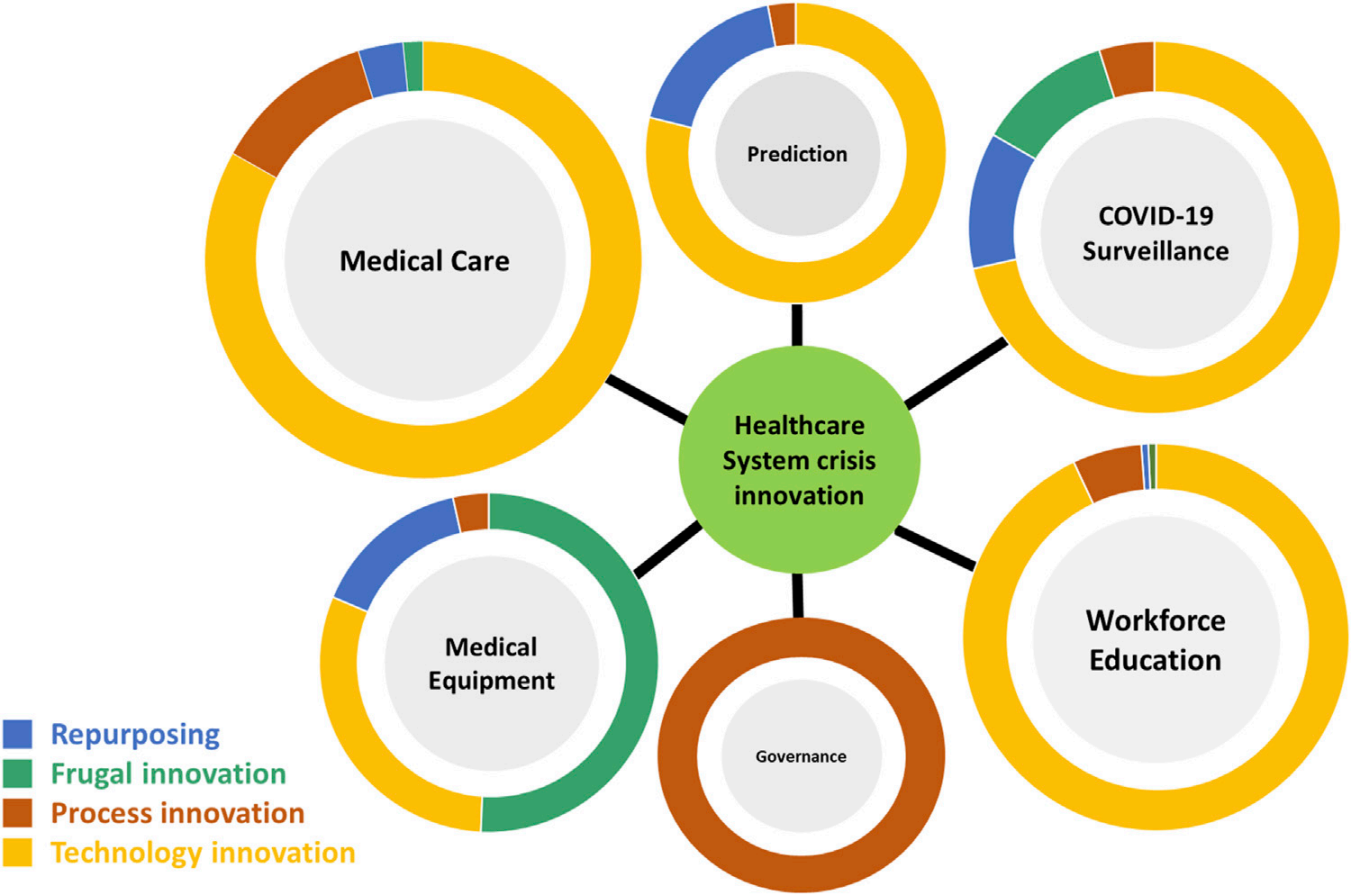
Healthcare system crisis innovation: Types of innovations in the six thematic categories (Taiwan, 2021).

The types of innovation during the healthcare system crisis across the six thematic categories are presented in Figure 5.

### Medical Care

Technological innovation, followed by process innovation, is the primary type of innovation in medical care. The use of technologies such as telemedicine, mobile applications, and wearable devices became a new norm that allowed specialists to monitor and manage patients remotely, including evaluation of personal details such as the course of illness, vital signs, and medication status [19]. Many technology-based approaches, including big data analysis and AI, were used to analyze patients’ clinical conditions, costs, and satisfaction to help health providers identify promising links between needs and solutions and generate new patient-centered care models [20]. In summary, most technological responses in medical care require more resources, but some of them seem to have the potential grow beyond the crisis. Moreover, new care processes and guidelines were maintained to simultaneously provide services and maintain social distancing.

### Medical Equipment

The main type of innovative responses in medical equipment involved innovations for frugality, which were characterized by a short response period and low resource requirements*.* The shortage of medical supplies and resources during the pandemic left patients and health providers unprotected, delayed time of diagnosis, and even delayed treatment [21]. Ready availability and low cost are the first consideration in settings with limited resources [22]. For instance, intubation boxes are used to ensure the protection of healthcare providers [23]. Health providers had no choice but to adapt to the situation and innovate using available resources and methods to continue their daily practice. In addition, the reuse of idle resources was a response to the rising demand for resources; for example, in the case of ventilators, while traditional production lines were accelerated, many firms outside the medical field also repurposed their production lines to make medical supplies [24, 25].

### COVID-19 Surveillance

Responses including technological innovations, innovations for frugality, and repurposing were mainly evident in COVID-19 surveillance. With the implementation of strict stay-at-home measures and travel restrictions, mass testing, rapid diagnosis, and identification became major priorities in many countries [26, 27]. In the early stage of the crisis, the innovative drive-through sampling approach in Korea, an easy idea to increase testing capacity and reduce costs, was later adopted by many countries to prevent cross-infection between testers in the waiting area [28]. Because of the limited diagnostic tools and high-risk environments, ultrasound was repurposed to facilitate clinical decision-making during triage, and chest CT combined with AI was used to accelerate diagnostic protocols [29]. Mobile applications and wearable devices were used for contact tracing and to ensure adherence to quarantine measures [30]. A variety of tools and technologies proposed worldwide have been innovated to break the chain of transmission [31].

### Workforce Education

The main type of innovative response in workforce education was technological innovation, which requires more resources and has the opportunity to become a new normal. Virtual learning, which shows a primary connection with “education” in a network graph, through technical innovations and applications such as online social media platforms allowed trainees to be supervised and evaluated as they continued to learn medical knowledge and skills remotely. Moreover, some techniques, such as virtual reality (VR) and simulation techniques, played a key role during the pandemic. Health educators adapted and rapidly responded to adopt novel means of teaching and assessment, which may improve medical education in the long-term [32].

### Prediction and Application

The main type of innovative response in prediction and application was technological innovation, which requires more resources and may have a longer response timeframe. While the world experienced a sharp growth in COVID-19 cases, an immense volume and variety of information regarding COVID-19 continued to accumulate. One promising breakthrough was the application of big data analytics and AI. Data science was used to project resource demands and predict hotspots by modeling the COVID-19 pandemic [33]. Some social media platforms and communication tools were also used to predict the risk and strengthen surveillance for new outbreaks.

### Governance

Process innovations predominated the governance category. The resource requirements and timeframes were changeable and variable, depending on the scale of the response and aspect of influence. In the early stages of COVID-19, healthcare providers and organizations were generally unprepared to deal with crises and manage the overloaded healthcare system [34]. Thus, the increased role of rolling wave planning, multisectoral cooperation, and interdisciplinary collaboration ensured a rapid response to the unpredictable healthcare crisis [11]. To this end, healthcare providers tried to identify better, cheaper, and safer innovations to improve the quality of care, services, and internal operations.

## Discussion

This section discusses the four types of innovative responses extracted: frugal innovation, repurposing, process innovations, and technological innovations.

### Frugal Innovation

Frugal innovation are aimed at delivering fundamental needs and providing effective solutions in low-resource settings [6, 26]. These innovations are based on two major models: one simplifies existing high-tech tools and reduces costs, and the other uses low-cost, low-tech, and even no-tech solutions to solve problems [6]. In a resource-constrained pandemic, the latter model is more common, since existing affordable methods or products do not satisfy the tremendous demands imposed on the healthcare system.

The COVID-19 crisis has forced us to re-examine neglected types of innovation in medicine [6]. Most of the innovations for frugality were short-term solutions that addressed the immediate challenges during the crisis. Such innovations do not necessarily imply low quality; however, they provide safe and effective healthcare given the circumstances and resource constraints. Therefore, the features above are valuable since first-line health providers can learn and imitate them quickly to solve clinical problems in times of low resource availability. The government and social media should help advocate the idea of innovations for frugality that can solve critical problems with low cost and low-tech responses, especially in the early stages of the pandemic. However, this approach still has limitations and is associated with safety considerations because most clinical physicians lack background knowledge of the medical device innovation process [22].

### Repurposing

Repurposing is a solution to meet the urgent innovation needs imposed by time and resource constraints. The three major fields of repurposing are as follows (1): tool repurposing for diagnosis (2) drug repurposing for treatment, and (3) manufacturing repurposing for medical equipment. The key advantages of drug repurposing include shortening the timeline and decreasing the cost of development, thereby effectively relieving the urgent need for therapeutic interventions in resource-limited settings. Moreover, using idle manufacturing capacity, temporary and inexpensive strategies can offer life-saving products.

For the healthcare system, addressing the shortage of resources and equipment is a major priority, especially in the early stages of a pandemic. Given time and resource constraints, standard solutions fail to address this urgency, and the government should loosen the restrictions and regulations to promote repurposing-based responses in the field of drug approvals, vaccines, or diagnostic tools. In addition, the government could enhance and integrate the relationships among different industries that can provide redundant or idle production capacity or equipment to address the insufficiency of existing resources. Thus, repurposing is not a long-term strategy, and products that bypass testing and clinical trials should be an area of concern. Despite doubts about the effectiveness of these solutions and equipment, they can be considered to be better than nothing at all [25].

### Process Innovations

For most industries, the goal of process innovation is related to a firm’s pricing policy and costs [15]. However, the stakeholders of process innovation in healthcare systems include patients, healthcare providers, governmental organizations, and payers. Traditionally, clinical management or procedures are contingent on the provider’s preferences and attitudes in the healthcare system [20]. During crises, exploration of alternative methods enabled healthcare providers to respond to the crisis while meeting the needs of self-protection and social distancing, and maintaining the quality of healthcare services by rapidly remodeling their services and restructuring their organizations. The requirement for resources is adjustable and changes gradually depending on the scale, workface, and resource status of the organization. Excellent responses in reviews can be learned but not copied completely due to the variety in organizational composition, culture, and habits of stakeholders. Many roadmaps and team strategies have been provided worldwide to ensure that excellent innovations remain, while others are adjusted as the crisis evolves to a new normal [35].

### Technological Innovations

Most articles used technology or “technological innovation’’ or “digital innovation’’ to describe their innovations obtained by technological convergence to solve problems during the COVID-19 crisis [36–41]. Technological innovation is an important response in many fields of healthcare systems and may have more long-term effects after the crisis, but it also requires more resource and technical requirements during the pandemic.

Telemedicine is the primary innovative response and is used as a substitute for face-to-face interactions to enhance clinical care, health promotion, and disease prevention in many countries worldwide. Internet of medical things (IoMT) is a branch of internet of things (IoT) that combines traditional medical devices with traditional IoT, which can reduce healthcare spending, improve the efficient use of resources, and provide better care for patients [42]. Throughout the crisis, the innovative application of AI and machine learning also extended across multiple domains, including disease prediction, detection, diagnosis, monitoring, treatment, and medication and vaccine development [19, 29].

Evidence from this review suggests that the response of crisis forces in technological innovation is rapidly increasing and has even been integrated into the policy of response to support evidence-based decision-making [11]. Although technological applications require more resources, their impact and potential would be better than that of other responses when the technique is used comprehensively. As telemedicine changes the model, way of communication, and experience of care, post-pandemic patients may be more willing to try telemedicine and consider it essential for healthcare services, resulting in increased adoption and innovation of telemedicine [27]. Service providers, planners, and policymakers should take the opportunity to address the resistance to adoption and accelerate the promotion of telemedicine services. In addition, the prediction of trends and new outbreaks during a pandemic is essential for policy interventions and efficient allocation of medical resources [11]. The role of techniques such as AI and big data should be emphasized to show the value of the next breakout of disease. In the field of education, virtual learning, VR, and simulation play promising roles in the future beyond the pandemic, since they provide extremely realistic environments for students to learn from and have been proven effective in cognitive training and clinical decision-making [43]. Most of the ideas and development of innovative responses are expected to continue and even grow in the global healthcare system after the pandemic.

As the pandemic evolves, the lessons learned from prior experiences and the emerging innovative responses around the world are essential, but regional differences in basic infrastructure, technology development, socioeconomic resilience, population diversity, and resources should also be taken into account. Based on the experience in 2020, countries and policymakers should rapidly deploy innovative responses to improve outbreak prediction, facilitate surveillance and contact tracing, and even streamline medical care to become front-runners in the early stage of the pandemic. Although some poor or resource-limited countries may face difficulty in copying the innovative responses, especially technological innovations, applied in developed countries, innovations for frugality and repurposing may play a role in low-resource healthcare settings and remote regions. First-line healthcare providers also have the opportunity to gain inspiration from previous experiences and respond during times of resource exhaustion, especially with innovations for frugality. However, some innovative responses may raise ethical and legal concerns. It is difficult for healthcare providers and the government to ensure privacy protection, tracing security, and governance in such situations. In addition, the safety of repurposed products, drugs, and temporal production lines is also a matter of concern. With the stabilization of the pandemic, these innovative responses will be reexamined and remodeled. Some types of innovative responses, such as innovations for frugality, disappear spontaneously once the resources are sufficient, and some, such as telemedicine, will grow and develop.

### Limitations

This study used longitudinal data on innovations in healthcare systems. However, as the pandemic is still ongoing, the data on innovation features and models only represent the findings during the first year of the pandemic. Moreover, the methodological characteristics of narrative reviews present limitations because of the unavailability of critical appraisal or quality assessment tools. More innovative solutions may have been ignored or not published because of the impending collapse of the healthcare system in the early stages of the pandemic. In addition, selection bias may have occurred during the data extraction. Moreover, since recording and sharing of some types of innovation may be difficult, especially process innovations, which require a longer period to accommodate modifications, publication bias may have been present.

### Conclusion

The COVID-19 pandemic has undoubtedly caused substantial disruptions to the healthcare system and challenged organizations’ innovation and creativity around the world in a short period of time. We provided insights on the features and types of innovation during the first year of the COVID-19 pandemic to facilitate the dissemination and adaptation of innovative responses that have been identified to be successful in previous studies.

A common feature of innovations in a crisis is that they seem to reflect a critical demand focused on the short term rather than envisioning future market opportunities. Frugal innovation represent an important strategy, especially in the thematic category of medical equipment. Moreover, repurposing to cut costs and time has been proven to accelerate and expand the capacity of the healthcare system in resource-constrained conditions. Technological innovation is the main innovation model, in which both telemedicine and IoT are indispensable techniques.

The initial focus of the COVID-19 crisis was on rapid response and innovation to help people with limited resources. However, with the easing of the crisis, some innovative responses will fade out, while others will persist and even evolve, especially technological innovations. By viewing the pandemic as an opportunity for innovation, we can acquire inspiration from these experiences to better prepare for future crises.
